# Identification of *Riptortus* *pedestris* Salivary Proteins and Their Roles in Inducing Plant Defenses

**DOI:** 10.3390/biology10080753

**Published:** 2021-08-05

**Authors:** Hai-Jian Huang, Xiao-Tian Yan, Zhong-Yan Wei, Yi-Zhe Wang, Jian-Ping Chen, Jun-Min Li, Zong-Tao Sun, Chuan-Xi Zhang

**Affiliations:** State Key Laboratory for Managing Biotic and Chemical Threats to the Quality and Safety of Agro-Products, Key Laboratory of Biotechnology in Plant Protection of MOA of China and Zhejiang Province, Institute of Plant Virology, Ningbo University, Ningbo 315211, China; huanghaijian@nbu.edu.cn (H.-J.H.); 1911074036@nbu.edu.cn (X.-T.Y.); weizhongyan@nbu.edu.cn (Z.-Y.W.); 2011074048@nbu.edu.cn (Y.-Z.W.); chenjianping1@nbu.edu.cn (J.-P.C.); lijunmin@nbu.edu.cn (J.-M.L.)

**Keywords:** *Riptortus* *pedestris*, salivary protein, plant defense, reactive oxygen species

## Abstract

**Simple Summary:**

The bean bug, *Riptortus pedestris* (Fabricius) is a notorious pest of soybean crops in Asia. During the feeding process, the bug secretes a mixture of salivary components, which play critical roles in the insect–plant interactions. In the present study, a total of 136 salivary proteins were identified by transcriptomic and proteomic approaches. Among them, five proteins (RpSP10.3, RpSP13.4, RpSP13.8, RpSP17.8, and RpSP10.2) were capable of inducing cell death, reactive oxygen species (ROS) burst, and hormone signal changes, indicating the potential roles of these proteins in eliciting plant defenses. Our results provide a good resource for future functional studies of bug salivary effectors and might be useful in pest management.

**Abstract:**

The bean bug, *Riptortus pedestris* (Fabricius), is one of the most important soybean pests. It damages soybean leaves and pods with its piercing-sucking mouthparts, causing staygreen-like syndromes in the infested crops. During the feeding process, *R. pedestris* secretes a mixture of salivary proteins, which play critical roles in the insect–plant interactions and may be responsible for staygreen-like syndromes. The present study aimed to identify the major salivary proteins in *R. pedestris* saliva by transcriptomic and proteomic approaches, and to screen the proteins that potentially induced plant defense responses. Altogether, 136 salivary proteins were identified, and a majority of them were involved in hydrolase and binding. Additionally, *R. pedestris* saliva contained abundant bug-specific proteins with unknown function. Transient expression of salivary proteins in *Nicotiana benthamiana* leaves identified that RpSP10.3, RpSP13.4, RpSP13.8, RpSP17.8, and RpSP10.2 were capable of inducing cell death, reactive oxygen species (ROS) burst, and hormone signal changes, indicating the potential roles of these proteins in eliciting plant defenses. Our results will shed more light on the molecular mechanisms underlying the plant–insect interactions and are useful for pest management.

## 1. Introduction

Plants and herbivorous insects have been engaged in a long-term co-evolutionary arms race. Generally, insects, especially those with piercing-sucking mouthparts, inject saliva into plant tissues, while the injected saliva then acts as the biochemical interface that plays critical roles in food processing [1]. Upon secretion, the bioactive saliva has an array of functions from anchoring and lubricating stylets, digesting nutrients and plant cell components to modulating plant defenses [2,3,4,5]. The identification of saliva components is the first step in understanding their functions. In recent decades, with the development of omic technology, salivary proteins from some agriculturally important pests have been reported, including aphids [6,7,8], planthoppers [9,10], leafhoppers [11], whiteflies [12], and spider mites [13,14,15]. However, the current knowledge on the salivary components of herbivores in true bugs is still limited.

In response, plants have evolved sophisticated systems to cope with insect attack. Typically, the first step of defense in plants is the recognition of ‘non-self’ molecules known as damage-associated molecular patterns (DAMP), herbivore-associated molecular patterns (HAMP), or plant endogenous molecules activated by digestive enzymes, by plant pattern recognition receptors (PRRs) [16]. To date, a number of salivary elicitors that activate plant defenses have been identified. It is reported that salivary proteins Nl12, Nl16, Nl28, Nl43, NlMLP, NlSP1, and LsPDI1 from planthoppers can be recognized by plants and elicit an array of defense responses, such as reactive oxygen species (ROS), cell death, callose deposition, and defense-related gene expression [17,18,19,20]. Moreover, salivary proteins Mp10, Mp42, Mp56, Mp57, and Mp58 from *Myzus persicae* have negative effects on *M. persicae* reproduction, which is likely achieved by activating the plant defense responses [21,22]. However, to the best of our knowledge, no salivary effector from true bugs has been reported to elicit plant defenses. Therefore, it is interesting to investigate the interactions between plant and true bugs via a salivary approach.

Apart from eliciting plant defenses, saliva also contains bioactive components that suppress host defenses. For instance, the glucose oxidase from *Helicoverpa zea* is the best known salivary protein that promotes insect feeding by inhibiting nicotine production and H_2_O_2_ generation [23,24]. For herbivores with piercing-sucking mouthparts, the secretion of some salivary proteins is also critical to modulate plant defenses. For example, the salivary macrophage migration inhibitory factor promotes aphid performance through inhibiting major plant immune responses, like callose deposition and hypersensitive cell death [25]. Besides, salivary DNase II assists in the colonization of planthopper on host plants by degrading DAMPs released from damaged cells [26]. Recently, mirid bug was found to secrete a glutathione peroxidase that eliminates ROS accumulation and inhibits the pattern-triggered, immunity-induced cell death [27]. Such result indicates that salivary proteins are also important for true bugs. The salivary components vary across different insect species [3]. Investigating the functions of salivary proteins will gain more insights into the insect–plant interactions and promote the development of novel strategies for pest control.

The bean bug, *Riptortus pedestris* (Fabricius), is a notorious pest of soybean crops in Asia. It absorbs nutrients from soybean leaves, pods, and seeds, resulting in a significant yield loss. In recent years, *R. pedestris* has received increasing attention due to its potential of causing soybean staygreen-like syndromes or *Zhengqing* (the Chinese common name), which is typically characterized by a lack of timely leaf senescence and an increase in seed abortion [28,29]. Under laboratory conditions and in the field cage experiments, the soybean plants infested by *R. pedestris* display syndromes such as abnormal seeds, empty pods, and a lack of timely leaf senescence [30]. Insect saliva is considered as one of the most important factors that modulate plant physiology [31]. The staygreen-like syndromes in the infested plants may be correlated with some salivary components of *R. pedestris*, which deserves further investigation. In our previous work, we successfully assembled *R. pedestris* genome and annotated the protein coding genes [30]. However, it remains unknown about the precise *R. pedestris* proteins secreted into plants and their corresponding functions. This study identified the salivary proteins of *R. pedestris* by transcriptomic and proteomic approaches first of all. Then, the salivary proteins that potentially induced plant defenses were analyzed by in planta transient expression, and the roles of proteins that mediated insect–plant interactions were unveiled.

## 2. Material and Methods

### 2.1. Insect Strain

The *R. pedestris* used in this study was originally collected from a soybean *Zhengqing* field (33.7° N, 117.0° E) in Suzhou, China in 2019. The insects were reared on soybean plants (strain: Wandou 27) at 26 ± 0.5 °C with humidity of 50 ± 5% under a 16/8 h (light/dark) photoperiod.

### 2.2. Transcriptomic Analysis

The salivary glands (SGs) were collected from adult *R. pedestris* using a pair of forceps in a phosphate-buffered saline (PBS) solution (137 mM NaCl, 2.68 mM KCl, 8.1 mM Na_2_HPO_4_ and 1.47 mM KH_2_PO_4_, pH 7.4). The isolated SGs were immediately transferred to RNAiso plus (TaKaRa, Dalian, China) and the total RNA was extracted according to the manufacturer’s recommendations. Altogether two biological replicates were performed, with each containing 20 SGs. Thereafter, the RNA samples with high integrity and quantity proceeded for library construction and Illumina sequencing in Novogene (Beijing, China). Briefly, poly (A) + RNA was enriched by oligo (dT) magnetic beads, and fragmentation was performed with cations at 94 °C for 5 min. Afterwards, the extracted RNA was reverse transcribed using N6 random primers. After end-repairing and adaptor ligation, the sample was subjected to PCR amplification and purification to create a cDNA library with the QIAquick PCR purification kit (Qiagen, Hilden, Germany). Subsequently, the library was sequenced on an Illumina platform, and the output raw data were filtered to remove the low quality reads. Then, the clean reads were aligned to the reference genome using HISAT2 [32]. The transcripts per million (TPM) expression value of each gene was calculated by StringTie [33]. The TPM values of each gene from *R. pedestris* guts, fat body, muscles, carcass, testes, and ovaries have been reported in our previous work [30]. For the identification of SG-specific genes, the TPM value of each gene in SG was compared with those in the other six tissues, respectively. The gene–relative abundance (ratio) and *p*-value were calculated based on differential expression analysis embedded in DESeq2 [34]. Only genes with fold changes > 10 and *p*-value < 0.05 in all comparison groups were considered as SG-specific genes.

### 2.3. Proteomic Analysis

The SGs used for proteomic analysis were collected from adult *R. pedestris.* The 50 SGs were pooled into one sample, which was later lysed with lysis buffer that contained 100 mM NH_4_HCO_3_, 8 M Urea and 0.2% SDS, followed by 5 min of ultrasonication on ice. The lysate was subsequently centrifuged at 12,000× *g* for 15 min at 4 °C, and the supernatant was transferred to a clean tube. The sample was then reduced with 10 mM DTT for 1 h at 56 °C and alkylated with sufficient iodoacetamide for 1 h at room temperature in dark. Then, the sample was completely mixed with 4 volumes of precooled acetone under vortexing and incubated at −20 °C for at least 2 h. The sample was then centrifuged and the precipitation was collected. After washing twice with cold acetone, the pellet was dissolved with the dissolution buffer. For trypsin digestion, 3 μL of 1 μg/μL trypsin and 500 μL of 50 mM TEAB buffer were added, and the sample was mixed and digested at 37 °C overnight. Later, formic acid was added into the digested sample, and centrifuged at 12,000× *g* for 5 min at room temperature. The supernatant was slowly loaded to the C18 desalting column, washed with washing buffer (0.1% formic acid, 3% acetonitrile) thrice, and eluted with the elution buffer (0.1% formic acid, 70% acetonitrile). The eluent of sample was collected and lyophilized.

LC-MS/MS analysis was performed following the methods described below. Briefly, the lyophilized powder was dissolved in 10 μL solution A (100% water, 0.1% formic acid), and centrifuged at 14,000× *g* for 20 min at 4 °C; later, 1 μg of sample was injected into a home-made C18 Nano-Trap column (2 cm × 75 μm, 3μm). Peptides were separated in a home-made analytical column (15 cm × 150 μm, 1.9 μm) using the linear gradient elution. The separated peptides were analyzed by the Q Exactive HF-X mass spectrometer (Thermo Fisher, Bremen, Germany), with the ion source of Nanospray Flex™ (ESI) (Thermo Fisher, Bremen, Germany) at the spray voltage of 2.3 kV and the ion transport capillary temperature of 320 °C. The top 40 precursors with the highest abundance in the full scan were selected and fragmented by higher energy collisional dissociation (HCD) and analyzed in MS/MS.

Afterwards, the resulting spectra from each fraction were searched separately against the *R. pedestris* database [30] by the search engines: Proteome Discoverer 2.2 (PD 2.2, Thermo Fisher, Bremen, Germany). The search parameters were set as follows: the mass tolerance for precursor ion was 10 ppm and the mass tolerance for product ion was 0.02 Da. Carbamidomethyl was specified as fixed modifications, oxidation of methionine (M) as dynamic modification, and acetylation as N-Terminal modification in PD 2.2. At most 2 missed cleavage sites were allowed. In order to improve the quality of analysis results, the PD 2.2 software was used to further filter the retrieved results.

### 2.4. Bioinformatics Analysis

Blast2GO was utilized to annotate the salivary proteins. BGI WEGO (http://wego.genomics.org.cn/cgi-bin/wego/index.pl accessed on 2 October 2020) was adopted to classify proteins into three main categories (biological process, cellular component and molecular function). TBtool was employed to identify the enriched GO terms using the following formula: $P=1-\sum_{i=0}^{m-1}\left(\frac{\left(\begin{array}{c} M\\ i \end{array}\right)\left(\begin{array}{c} N-M\\ n-i \end{array}\right)}{\left(\begin{array}{c} N\\ n \end{array}\right)}\right)$, where *N* represents the number of gene with GO annotation, *n* represents the number of interested genes in *N*, *M* stands for the number of genes in each GO term, *m* indicates the number of tissue-specific genes in each GO term [35]. A value of *p* < 0.05 was considered significant enrichment. To analyze the proteins potentially secreted, the presence of signal peptides was firstly predicated by the SignalP 4.1 Server (http://www.cbs.dtu.dk/services/SignalP/ accessed on 2 October 2020). Then, proteins containing a signal peptide were searched against the TMHMM Server v. 2.0 (http://www.cbs.dtu.dk/services/TMHMM/ accessed on 2 October 2020), so as to identify the potential transmembrane domains. Proteins without or with one transmembrane domain included in the predicted signal peptide were considered as the secreted protein.

To identify the salivary proteins commonly identified in *R. pedestris* and other pierce-sucking insects, comparative analysis was performed by local BLAST alignment. The salivary proteins used as queries were collected from Sternorrhyncha species [6,7,8,12,36,37,38,39], Auchenorrhyncha species [9,10,40,41], Heteroptera species [42,43,44,45], and Lepidopteran species [46,47,48]. The proteins with high sequence homology (an E-value cut-off of 10^−5^) were further BLAST searched against the NCBI database. Only the proteins with similar annotation and high sequence similarity were grouped together.

### 2.5. In Planta Expression of Salivary Proteins

To investigate the roles of salivary proteins in inducing plant defenses, in planta expression of salivary proteins was performed in *Nicotiana benthamiana* leaves. Briefly, the coding sequences without signal peptides were amplified from cDNA of *R. pedestris* SG using the primers listed in Table S1. Then, the purified PCR products were cloned into binary vector that containing GFP using the Trelief^TM^ SoSoo Cloning Kit (Tsingke, Nanjing, China). The recombinant plasmids were subsequently introduced into *Agrobacterium tumefaciens* GV3101. Afterwards, the *A. tumefaciens* containing target plasmids were harvested by centrifugation and resuspended in the infiltration buffer (10 mM MgCl_2_, 10 mM MES, 150 mM acetosyringone). Infiltration was performed by injecting agrobacterium cell suspensions into *N. benthamiana* leaves using a needleless syringe. The plants were kept in a climate chamber under the temperature of 23 ± 1 °C, the RH of 70–80%, and the light/dark photoperiod of 16/8 h. At 48 h post-infiltration, the samples were collected for DAB staining and real-time quantitative PCR (qPCR) analysis.

### 2.6. DAB Staining

The ROS level in *N. benthamiana* was detected by DAB staining. In brief, the tobacco leaf was cut and immersed into 1 mg/mL DAB-HCl (pH 3.8, Sigma, St. Louis, MO, USA) for 6 h. Then, the DAB solution was replaced with 100% ethanol and decolored overnight at 65 °C. The stained sample was then photographed using a Canon EOS 80D camera (Canon Inc., Tokyo, Japan).

### 2.7. qPCR Analysis

The *N. benthamiana* samples were collected by a punch (8 mm in diameter) and homogenized in RNAiso plus. After extraction, the extracted total RNA was reverse transcribed using the HiScript II Q RT SuperMix Kit (Vazyme, Nanjing, China) to synthesize the first-strand cDNA. In this process, the contaminant genomic DNA was removed by adding gDNA remover in the kit. qPCR was performed using the SYBR Green Supermix Kit (Yeasen, Shanghai, China) with the Roche Light Cycler^®^ 480 Real-Time PCR System (Roche) by the following reaction program: denaturation for 5 min at 95 °C, followed by 40 cycles at 95 °C for 10 s and 60 °C for 30 s. The qPCR primers, which were designed by Primer Premier 6.0, are listed in Table S1. The housekeeping genes for actin was used as the internal control. The output data were calculated by a relative quantitative method (2^−∆∆Ct^) [49]. Statistical significance between GFP control and each salivary protein was calculated using Student’s *t* test. Three biological replicates were performed.

## 3. Results

### 3.1. Overview of the Riptortus pedestris Transcriptome

**Figure 1:**
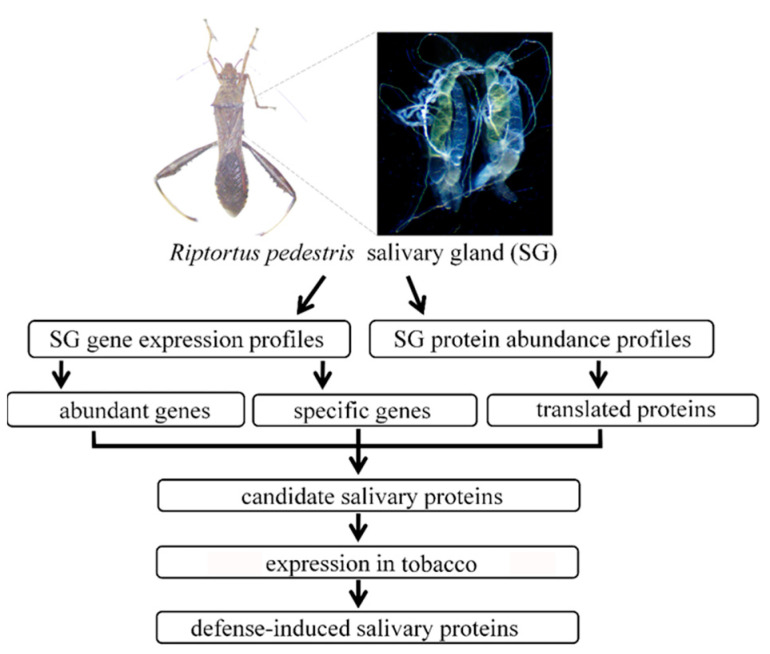
Overview of workflow used to identify the defense-induced salivary proteins. The salivary glands isolated from adult *Riptortus pedestris* were subjected to transcriptomic and proteomic analyses. The secretory proteins that were abundantly and specifically expressed in *R. pedestris* salivary gland were considered as salivary proteins. Then, the salivary proteins were transiently expressed in *Nicotiana benthamiana*, and the defense-induced salivary proteins that were capable of inducing cell death, reactive oxygen species (ROS) burst, or hormone signal changes were identified.

**Figure 2:**
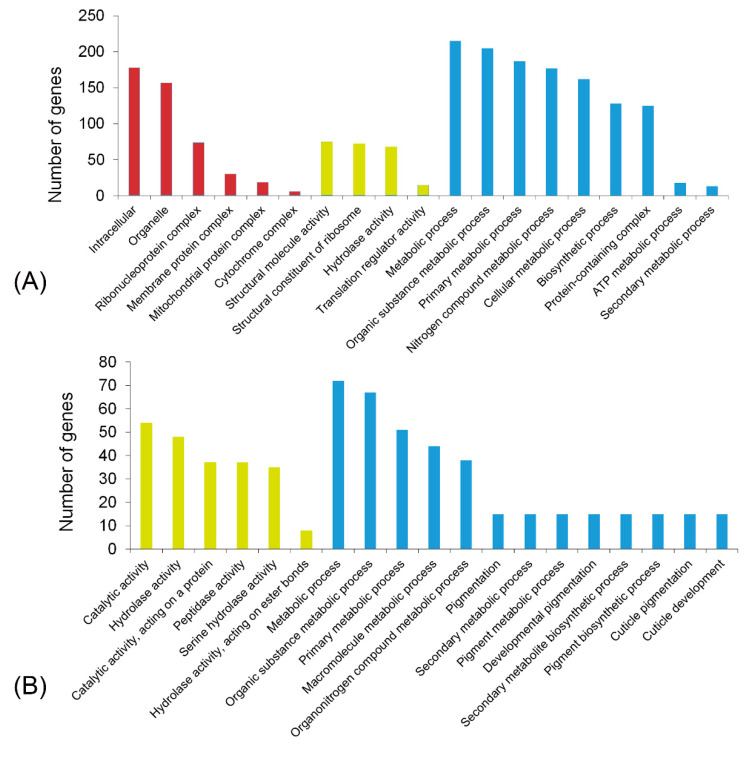
Function analysis of the interested genes. GO enrichment analysis on genes abundantly (**A**) and specifically (**B**) expressed in the salivary gland. A value of *p* < 0.05 was considered significant enrichment. Red column, cell component; orange column, molecular function; blue column, biological process.

The salivary proteins of *R. pedestris* were identified according to the workflow shown in Figure 1. For transcriptomic analysis, the library of SG was constructed and sequenced on the Illumina platform. After removing the low quality data, the clean reads were mapped to the reference genome, and then the expression level of each gene was determined. Altogether 8042 genes were validated to be expressed in SG with TPM > 1, which accounted for 42.3% of the predicated genes (Table S2). Among them, 456 genes were abundantly expressed with TPM > 100. Noteworthily, as many as 71 ribosomal proteins were abundantly expressed in SG, indicating the active protein synthetic process in this secretory tissue. In addition, 181 proteins were predicted to contain a signal peptide, which was indicative of the secretory property. Additionally, genes associated with hydrolase activity, including serine protease, amylase, lipase, and carboxypeptidase, were abundantly expressed in SG (Figure 2A; Table S2).

Many salivary proteins are specifically expressed in SG [12]. The transcriptome from *R. pedestris* guts, fat body, muscles, carcass, testes, and ovaries has been reported in our previous study. Here, this work investigated the SG-specific genes based on the transcriptomic data in our previous study [30] and the present study. A total of 226 genes were found to be specifically expressed in SG (Table S3). Gene ontology (GO) analysis demonstrated that genes associated with catalytic activity, hydrolase activity, and metabolism were significantly enriched (Figure 2B).

Moreover, Venn diagram analysis between SG-abundant genes and SG-specific genes was conducted, which identified 178 overlapping genes in two groups. Among these genes, 140 were predicated to contain a signal peptide, including four possessing at least one transmembrane domain besides the signal peptide. Such result indicated that these proteins might be embedded in the cell membranes of salivary glands. Taken together, there were 136 secretory genes that were abundantly and specifically expressed in *R. pedestris* SG (Table S4).

### 3.2. Overview of the Riptortus pedestris Proteome

The presence of salivary proteins in *R. pedestris* SG was investigated at the protein level by LC-MS/MS. A total of 775 proteins were detected (Table S5). Among them, 377 (48.6%) had binding functions, including ion binding, cofactor binding, protein binding, and lipid binding; 165 (21.3%) had regulatory functions, including metabolic regulation, signaling regulation, and developmental regulation; 266 (34.3%) were related to hydrolase, oxidoreductase, and lyase activity; whereas 39 (5.0%) were associated with transportation and transduction (Figure S1). Among the 136 secretory genes that were abundantly and specifically expressed in SG, 88 were found to be translated into proteins (Table S4). For the other 53 genes, no protein was detected, which might be caused by the insensitivity of LC-MS/MS analysis, protein degradation, or the failure to translate these genes, and further investigation was needed.

### 3.3. Characteristics of Salivary Proteins

**Figure 3:**
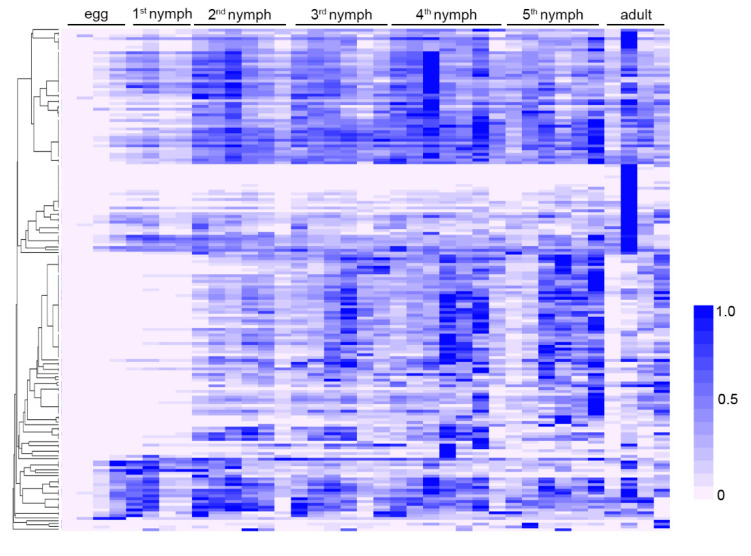
Expression profile of salivary proteins at different developmental stages. The relative expression levels of 136 salivary proteins at egg, nymph, and adult stages were calculated based on the transcriptomic data and illustrated by a heat map. The insects used for the developmental analysis were reared on soybean plants.

First of all, this study investigated the developmental expression patterns of 136 predicted salivary proteins. The insects (reared on soybean plants) of 37 different development stages were analyzed. It was found that all the salivary proteins were expressed at the lowest level during the egg periods (Figure 3). This is the typical expression pattern for most salivary proteins, as the insect does not need to feed before hatching [50]. Most salivary proteins were stably expressed in nymph and adult periods. However, yellow-like and four unannotated proteins (Rp.chr2.1812, Rp.chr3.1239, Rp.chr3.1227, Rp.chr1.0390) were highly expressed in the adult period. It will be interesting to investigate their specific functions in the adult period.

Interestingly, it was found in this study that salivary proteins were unevenly distributed in the genome. More than one half of salivary proteins were located on chromosome 1. In contrast, the sex chromosome did not contain any salivary protein. Additionally, 95 salivary proteins contained two or more amino acid tandem repeats (Table S4). For example, the seven serine rich proteins, with a homologous gene only found in *Oncopeltus fasciatus*, were tandemly arrayed. Similar phenomena were also observed in venom serine protease, carboxypeptidase, and some *R. pedestris*-specific genes, indicating that the salivary genes underwent expansion during the long-term evolution process.

All of the *R. pedestris* salivary proteins were compared with those from 24 other arthropod species (Table S6). Altogether, seven gene families were found to be commonly identified between *R. pedestris* and other arthropod species, including serine protease, lipase, carboxypeptidase B, protein yellow, amylase, inositol polyphosphate phosphatase, and lectin. Besides, up to 86 proteins were specifically identified in *R. pedestris* or other evolutionarily related bug species (Table S4). These proteins with unknown function have not been investigated in previous studies. However, their specific roles in *R. pedestris* and host plant interactions remain further investigation.

### 3.4. Function Analysis of Salivary Proteins

**Figure 4:**
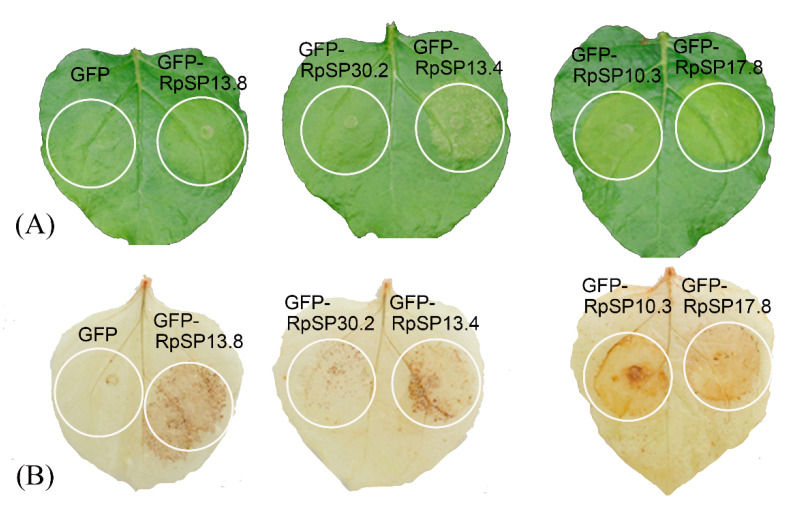
Salivary proteins induce cell death and reactive oxygen species (ROS) burst in *Nicotiana benthamiana*. (**A**) Five salivary proteins fused with the green fluorescent protein (GFP) were transiently expressed in *N. benthamiana* by agroinfiltration. Pathology symptoms were photographed 2 days post-infiltration. (**B**) DAB staining of the *N. benthamiana* transiently expressing salivary proteins. Tobacco leaves expressing GFP only were used as negative controls.

Cell death and ROS burst are considered to play critical roles in the plant defense responses induced by salivary components. To identify the candidate salivary proteins capable of eliciting plant defense signals, the salivary proteins were transiently expressed in *N. benthamiana* by agrobacterium infiltration. Five salivary proteins were found to be capable of inducing cell death, chlorosis, or ROS burst (Figure 4). As these proteins were bug-specific with unknown functions, we temporarily annotated them as RpSP10.3, RpSP13.4, RpSP13.8, RpSP17.8, and RpSP30.2 (RpSP is abbreviation of *Riptortus pedestris* salivary protein) based on their molecular weights.

**Figure 5:**
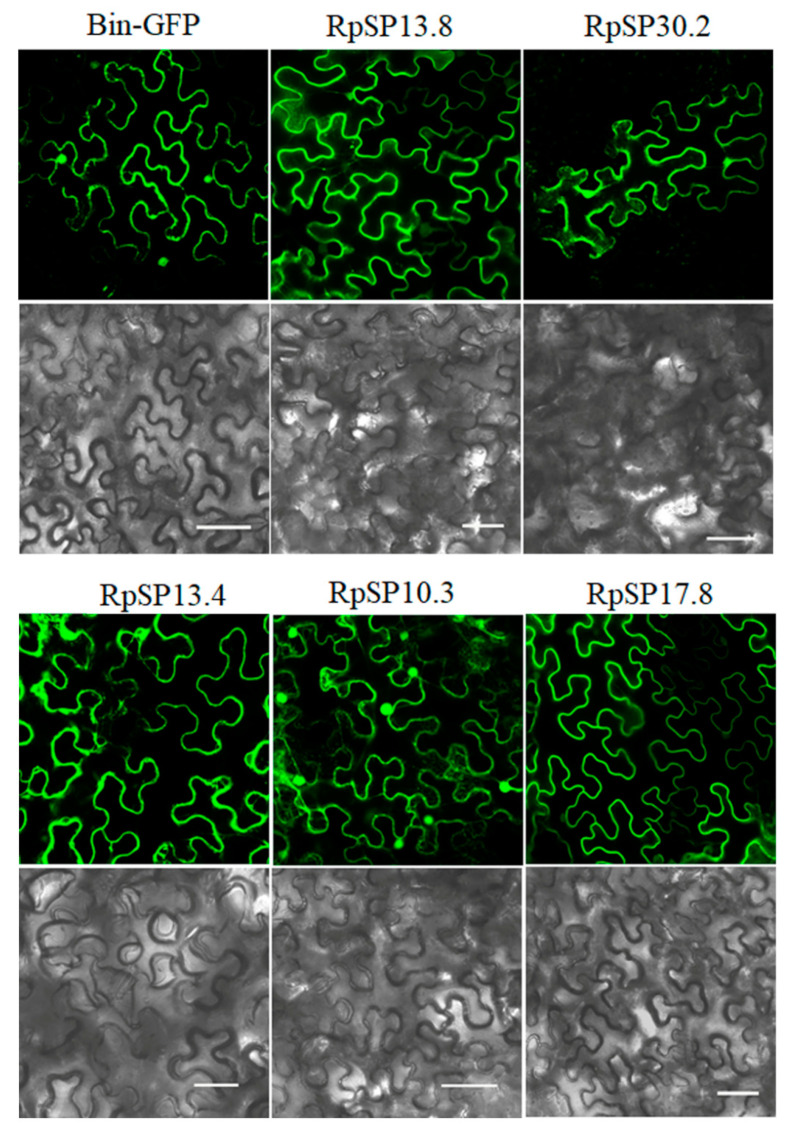
Subcellular location analysis of salivary proteins in *Nicotiana benthamiana*. Salivary proteins fused with the green fluorescent protein (GFP) were transiently expressed in *N. benthamiana* by agroinfiltration, and the fluorescence images were examined using a Leica confocal laser-scanning microscope SP8. Bars: 50 μm.

**Figure 6:**
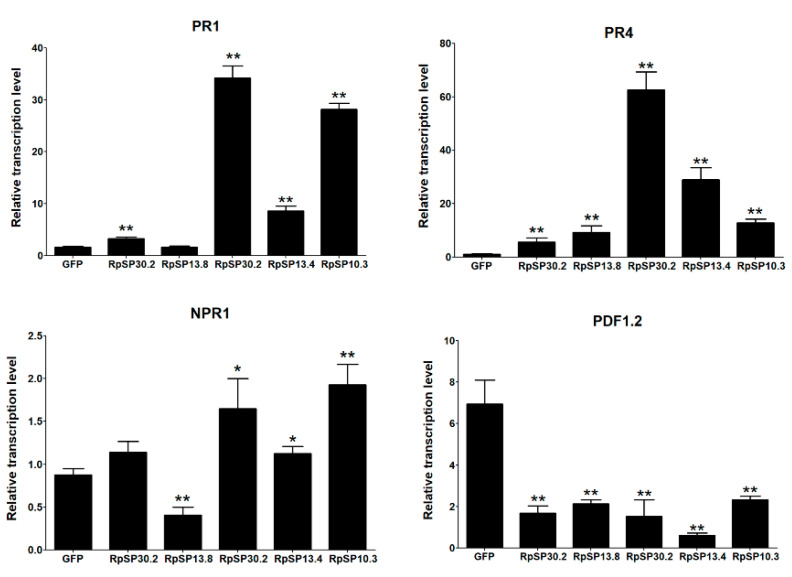
Salivary proteins activate the plant defense responses and alter plant hormone signals. Salivary proteins were transiently expressed in *N. benthamiana* by agroinfiltration. The relative transcript levels of PR1, PR4, NPR1, and PDF1.2 were determined by qPCR. Tobacco leaves expressing GFP only were used as negative controls. Statistical significance (Student’s *t* test) between GFP control and each salivary protein was calculated, respectively. * indicates *p* < 0.05, ** indicates *p* < 0.01. Bars: ±standard errors (SE).

The RpSP13.8 and RpSP13.4 induced severe cell death and chlorosis in *N. benthamiana*. DAB staining demonstrated that both proteins were capable of inducing ROS burst in plants, with RpSP13.8 inducing the strongest ROS burst. Subcellular location analysis of the RpSP13.8 and RpSP13.4 proteins fused with the green fluorescent protein (GFP) showed that both proteins located specifically in the cytoplasm (Figure 5). Pathogenesis-related proteins are induced by the plants as a defense response system in stress conditions like microbial and insect infections [51]. Both RpSP13.4 and RpSP13.8 induced significant upregulation of pathogenesis-related protein (PR1) and PR4 (Figure 6). Plant hormone regulatory networks, especially the jasmonate (JA) and salicylic acid (SA) pathways, sophisticatedly responded to insect attacks [52]. We found that the JA-responsive gene PDF1.2 was significantly repressed in *N. benthamiana* transiently expressed with RpSP13.4 and RpSP13.8. In contrast, the SA-responsive gene NPR1 was significantly upregulated in RpSP13.4-expressed leaves when compared with that of GFP control (Figure 6). Chlorosis phenotypes were observed in *N. benthamiana* transiently expressed with RpSP10.3 and RpSP17.8. These two proteins also induced ROS burst and the expression of PR1 and PR4. RpSP10.3 was mainly expressed in both the cytoplasm and the nucleus, while RpSP17.8 was specifically expressed in the cytoplasm (Figure 5). A weaker, but significant ROS burst was observed in *N. benthamiana* transiently expressed with RpSP30.2. RpSP30.2 also induced the expression of PR4 and NPR1. In contrast, the expression of PDF1.2 was significantly repressed in RpSP30.2-expressed leaves (Figure 6).

## 4. Discussion

Saliva contains a mixture of bioactive molecules that play critical roles in mediating the insect–plant interactions. In this study, the salivary proteins from *R. pedestris* were identified by transcriptomic and proteomic approaches. It was found that a majority of salivary proteins were tandemly arrayed in the genome and showed low expression in the egg stages. Except for proteins involved in hydrolase and binding, many salivary proteins were bug-specific or even *R. pedestris*-specific, and some of them were capable of inducing plant defenses.

Salivary proteins associated with hydrolase were significantly enriched, including serine protease, trypsin, carboxypeptidase, lipase, nuclease, and amylase. The bean bug uses the soybean seeds and plants as its main food sources, and the protein/oil-rich seeds are necessary to complete the insect life cycle [53]. Proteases and lipases are possibly secreted to digest proteins and oils in seeds. Previously, extra-oral digestion is generally presumed to be critical for carnivorous insects, which can secrete trypsin, chymotrypsin, and other digestive enzymes to decompose solid contents into liquid and increase the food extraction efficiency [54]. In recent years, the extensive identification of protease, amylase, and lipases in herbivorous saliva indicates that extra-oral digestion is also applied for herbivorous insects [55]. However, the abundance of hydrolase in insect saliva varies across species. As for planthopper that mainly depends on plant phloem saps, no trypsin is specifically expressed in SG, and the insect secretes less amounts of proteases during feeding [56]. For *R. pedestris*, abundant trypsins are specifically expressed in SG at high levels, indicating that the secretion of proteases may be more important for bugs during feeding. In rearing *R. pedestris*, the bug can smoothly pierce and suck dry the soybean seeds. It was presumed in this study that *R. pedestris* secreted abundant hydrolases to break down the solid proteins, oils, and amyloses into liquid, and then absorbed this liquified nutrition.

There were 86 proteins specifically identified in *R. pedestris* or its evolutionarily related bug species. Although these species-specific proteins have not been well characterized yet, their functions in *R. pedestris* and plant interactions cannot be ignored. During insect feeding, the secreted saliva intimately interacts with host plants, and the compositions can be easily shaped by natural selection [48]. For the time being, many salivary effectors identified are found to be species-specific. In *Nilaparvata lugens*, three planthopper-specific proteins (NlSHP, salivap3, and NlMul) are found to be indispensable for insect feeding on rice plants [9,18,50]. In *Acyrthosiphon pisum*, the critical salivary effectors (C002 and ACYPI009881) are limited to aphid species [21,57,58]. The species-specific salivary protein is possibly the result of the co-evolutional arm race between insects and plants. For example, the whitefly-specific salivary effector Bt56 attenuates plant defense responses by targeting plant NTH202 [59]. Further, the aphid-specific salivary effector Mp1 increases insect virulence by interacting with plant VPS52 [60]. The soybean staygreen-like syndromes induced by *R. pedestris* infection are significantly different from plant syndromes caused by aphids or whiteflies. Staygreen-like syndromes may be correlated with some bug-specific salivary proteins, which deserves further investigations.

Herbivore-associated molecular patterns derived from saliva have been documented to activate an array of plant defenses against herbivores, including ROS burst, mitogen-activated protein kinase (MAPK) cascade activation, and hormone signal transduction [1,31]. This study is the first to screen the bug-specific salivary components that potentially initiate plant defense responses. According to our results, transient in planta expression of five proteins (RpSP10.3, RpSP13.4, RpSP13.8, RpSP17.8, and RpSP30.2) induced ROS burst in *N. benthamiana*. Generally, rapid and transient accumulation of ROS is a common strategy for plants to cope with biotic and abiotic stresses [61,62]. It is the first line of barrier against subsequent attack by pathogens and herbivores, which acts as the secondary messengers to control defense responses [61,62]. Therefore, cell death and alteration in the hormone pathways of transfected plants may be positively correlated with ROS burst [20]. Although the five proteins are found to induce plant defenses, there may be other salivary components that suppress these detrimental effects, such as macrophage migration inhibitory factor in *M. percicae*, [25] and Al6 in *Apolygus lucorum* [27]. Further studies are warranted to unveil the complex mechanisms of *R. pedestris* saliva in insect–plant interaction.

## 5. Conclusions

This study analyzed the salivary components of *R. pedestris* by transcriptomic and proteomic approaches. Altogether, 136 secretory genes were discovered to be abundantly and specifically expressed in SG. Besides, it was found that salivary proteins associated with hydrolase potentially contributed to extra-oral digestion, while the bug-specific salivary proteins might be correlated with long-term *R. pedestris*–plant interaction. Among the bug-specific proteins, RpSP10.3, RpSP13.4, RpSP13.8, RpSP17.8, and RpSP30.2 were identified as the potential salivary effectors that induced plant defenses. The salivary secretome of *R. pedestris* described in this study helps to understand the molecular mechanisms underlying plant–insect interactions and provides valuable resources for further research.

## Data Availability

The data presented in this study are available to public. Raw reads generated by transcriptomic sequencing was submitted to the NCBI Sequence Read Archive under accession number: PRJNA671796. The raw data generated by LC-MS/MS analysis was submitted to Zenodo (https://zenodo.org/ accessed on 2 October 2020) with the http://doi.org/10.5281/zenodo.5112678.

## Supplementary Materials

The following are available online at https://www.mdpi.com/article/10.3390/biology10080753/s1, Figure S1: Function analysis of proteins detected in salivary glands. The proteins detected in salivary glands were classified into cell component (red), molecular function (orange), and biological process (blue), respectively. Table S1: Primers used in this study. Table S2: The *Riptortus pedestris* genes expressed in the salivary glands. Table S3: Expression patterns of SG-specific genes in different *Riptortus pedestris* tissues. Table S4: The salivary proteins of *Riptortus pedestris*. Table S5: The *Riptortus pedestris* proteins in salivary glands by LC-MS/MS. Table S6: Salivary proteins commonly identified in different arthropod species.
