# Supplementary material for: Identification of Riptortus pedestris Salivary Proteins and Their Roles in Inducing Plant Defenses
Source: Biology (Basel). 2021 Aug 5;10(8):753. doi: 10.3390/biology10080753 (PMC8389542; doi:10.3390/biology10080753)
Supplement: Supplementary file 1 [file biology-10-00753-s001.zip › Table S1.pdf]

Table S1 Primers used in this study

Gene	Forward primer (5'-3')	Reverse primer (5'-3')
**Primer used for plasmid construction**		
RpSP13.8	ACGAGCTGTACAAGGGTACCATGAAATATCCCAAGACAGCTTGC	GCGGACTCTAGTTCATCTAGATTATCTTCCGTAGATTGTAATA
RpSP30.2	ACGAGCTGTACAAGGGTACCATGGAACCTCTGCGCCAGAAAAGG	GCGGACTCTAGTTCATCTAGATTATGCGGCAGCTTGAGGCCT
RpSP13.4	ACGAGCTGTACAAGGGTACCATGTTCACGAGCCTCATCAAAGCG	GCGGACTCTAGTTCATCTAGATTAAATTCCCAGACTCAGCCTG
RpSP10.3	ACGAGCTGTACAAGGGTACCATGGAGCCTTTGTTTCTGAAGCGTG	GCGGACTCTAGTTCATCTAGATCAGCAGTCATCTTCGCATTC
RpSP17.8	ACGAGCTGTACAAGGGTACCATGCAGAGAAATACCATTGACAATGC	GCGGACTCTAGTTCATCTAGATTAACCTTGTGGTGATGGTCCC
**Primer used for qPCR**		
NPR1	TGCTCTCCATTATGCTGTAG	GCACTGTGTATCCTCTTGAA
PDF1.2	TGGCAAAATCTATGCGCTTT	ATCCTTCGGTCAAACAGACG
PR1	TGAGATGTGGGTCGATGAGA	CGAGTTACGCCAAACCACTT
PR4	GGCCAAGATTCCTGTGGTAGAT	CACTGTTGTTTGAGTTCCTGTTCCT
actin	GGAGAAGTTGGCTTACATTG	TCATTGATGGTTGGAACAGA
