# Supplementary figures and images for: Identification of Riptortus pedestris Salivary Proteins and Their Roles in Inducing Plant Defenses
Source: Biology (Basel). 2021 Aug 5;10(8):753. doi: 10.3390/biology10080753 (PMC8389542; doi:10.3390/biology10080753)

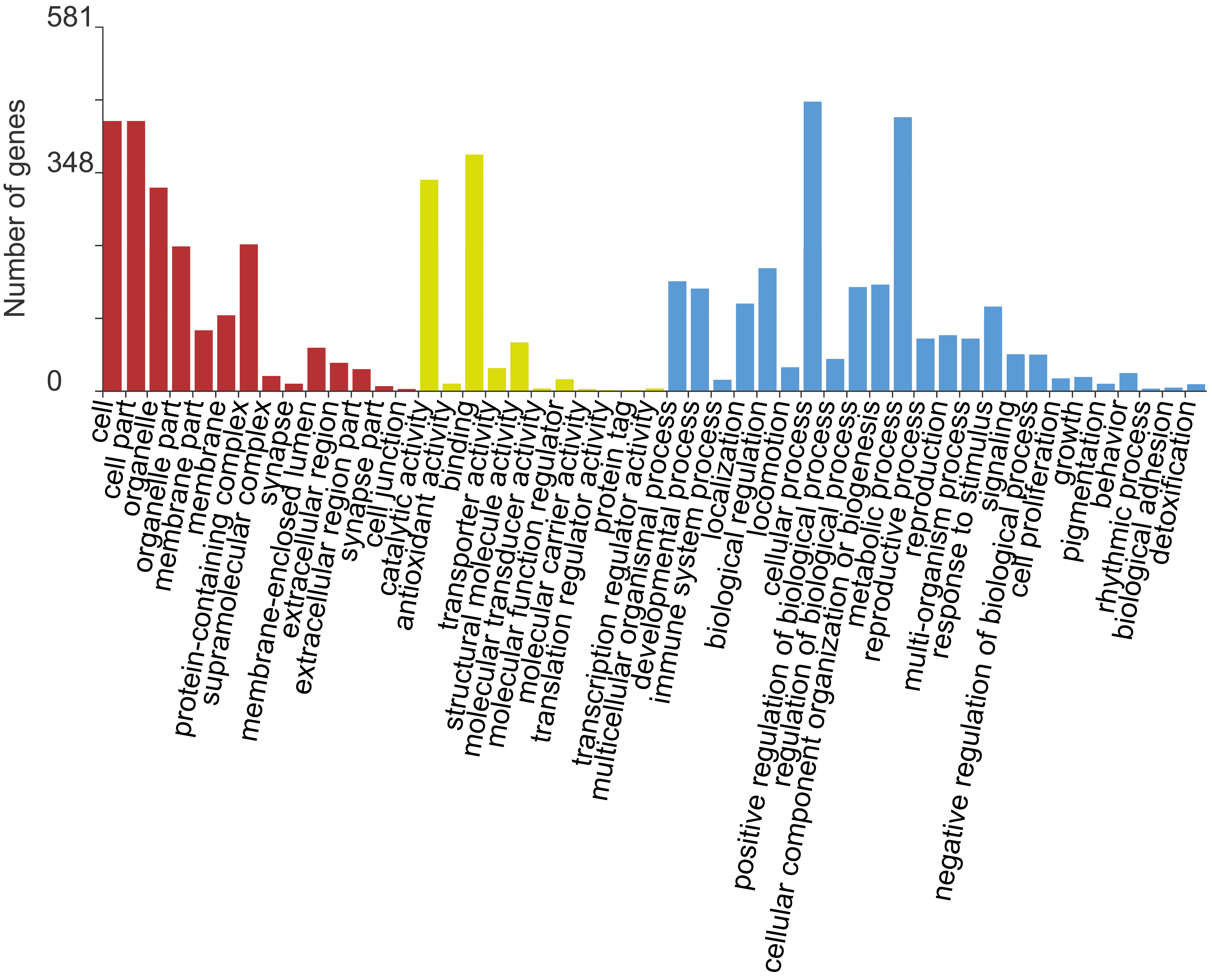

Supplement: Supplementary file 1 [file biology-10-00753-s001.zip › Figure S1.tif]
